# An ensemble model based on transfer learning for the early detection of Alzheimer’s disease

**DOI:** 10.1038/s41598-025-22025-y

**Published:** 2025-10-03

**Authors:** Zahra Asghari Varzaneh, Seyyed Mohammad Mousavi, Reza Khoshkangini, Sayyed Mostafa Moosavi Khaliji

**Affiliations:** 1 Department of Computer Science and Media Technology, Sustainable Digitalisation Research Center, Malmö University, Malmö, Sweden; 2 Health Information Sciences Department, Faculty of Management and Medical Information Sciences, Kerman University of Medical Sciences, Kerman, Iran; 3 Department of Research and Technology Activities Support of Kerman Provincial Unit, University of Applied Science and Technology, Tehran, Iran

**Keywords:** Alzheimer’s disease, Convolutional neural network, Transfer learning, Medical imaging, Ensemble learning, Neuroscience, Diseases, Medical research

## Abstract

Alzheimer’s disease (AD) is a progressive neurodegenerative disorder characterized by the gradual decline in cognitive functions, particularly memory and reasoning. Early detection, especially during cognitive impairment (MCI) stage, is crucial for timely intervention and management. Enhanced diagnostic methods are essential for facilitating early identification and improving patient outcomes. This study presents a robust deep learning framework for the early detection of Alzheimer’s disease. It employs transfer learning and hyperparameter-tuning of InceptionResnetV2, InceptionV3, Xception architectures to enhance feature extraction by leveraging their pre-trained capabilities. An ensemble voting mechanism has been integrated to combine predictions from different models, optimizing both accuracy and robustness. The proposed ensemble voting approach demonstrated exceptional performance, achieving 98.96% accuracy and 100% precision for predicting classes Mildly Demented and Moderately Demented. It outperformed baseline and state-of-the-art models, highlighting its potential as a reliable tool for early diagnosis and intervention.

## Introduction

 Alzheimer’s disease (AD) is the most common form of dementia, a term that refers to the decline in memory and other cognitive abilities, which can significantly impact a person’s daily life^1–4^. Typically, Alzheimer’s begins with mild cognitive impairment (MCI), which progressively worsens over time^5–7^. Individuals with MCI are at a higher risk of developing AD compared to the general population. AD is a complex and progressive neurological disorder that leads to the gradual deterioration of brain cells, ultimately affecting a person’s memory, thinking skills, and cognitive functions^8–10^. In the advanced stages of the disease, patients may experience severe weaknesses and a decline in the functionality of vital bodily systems. This decline can lead to secondary complications over time, ultimately resulting in mortality^8,11^.

Currently, the most reliable method for diagnosing Alzheimer’s dementia involves monitoring individuals with MCI and evaluating their cognitive changes over the years^12,13^. Magnetic resonance imaging (MRI) can detect alterations in brain structures that indicate the onset of AD. Early diagnosis of MCI due to Alzheimer’s is crucial, as individuals may benefit from early interventions aimed at preventing or delaying cognitive decline^14–16^. Typically, a neurologist will ask about the patient’s symptoms and medical history, as well as that of their immediate family, while also evaluating for other neurological disorders. To confirm a diagnosis of dementia, a series of diagnostic tests is required. These may include brain imaging to rule out other similar conditions, CT scans and MRIs to assess brain issues related to strokes or tumors, and PET scans for a more detailed examination of brain activity^17^. Early identification of AD, along with preventive measures, can help slow the progression of the disease and improve the quality of life for those affected^18,19^.

Early detection of AD is essential for effective treatment and improved patient outcomes. Traditional diagnostic methods typically rely on neuropsychological assessments and clinical evaluations, which can often be time-consuming. In recent years, the integration of artificial intelligence (AI) algorithms, particularly machine learning (ML) and deep learning (DL) techniques, has transformed disease diagnosis, providing greater accuracy and efficiency^20–23^. AI algorithms have shown significant promise in analyzing complex medical data. While machine learning models have been widely adopted, deep learning models, especially convolutional neural networks (CNNs), have demonstrated superior performance in various medical applications^24–27^. These models excel at automatically extracting hierarchical features from raw data, minimizing the need for extensive manual preprocessing. In the field of medical imaging, deep learning techniques have significantly enhanced the analysis of images, such as magnetic resonance imaging (MRI) and computed tomography (CT) scans. CNNs are particularly valuable because they can identify patterns and structures within these images, aiding in the diagnosis of numerous medical conditions^28,29^. In the context of Alzheimer’s disease, CNNs can extract critical features from MRI scans to help identify early signs of neurodegeneration by analyzing brain structures, including the hippocampus^30–34^. As research progresses, the potential applications of AI in medical diagnosis will continue to grow, paving the way for more effective healthcare solutions.

Venugopalan et al.^35^ classified patients into three groups: control (CN), mild cognitive impairment (MCI), and AD. They did this by analyzing several sorts of data using deep learning algorithms. They applied 3D-CNN to the imaging data and stacked denoising autoencoders to the clinical and genetic datasets for feature extraction. They also presented a novel method for interpreting data that uses perturbation analysis and clustering to identify the best characteristics found by deep learning models. Results show that deep learning models perform noticeably better than conventional shallow models. Helaly et al.^36^ introduced a comprehensive method for early AD identification that makes use of CNNs and deep learning. The first approach used both 2D and 3D convolutional approaches to process 2D and 3D structural brain scans from the ADNI dataset using simple CNN architectures. In order to use pretrained models, such VGG19, for medical picture classification, the second technique used transfer learning. Lin et al.^37^ employed the method of coupling a pre-trained 2D CNN for transfer learning with a reliable relational vector machine for the regression approach, rather than a 3D CNN. Feature transfer learning, 3D feature concatenation, and dimensionality reduction are important techniques used in this study. To estimate brain age, sMRI data is used to train the model. They proposed a pre-trained AlexNet for feature extraction, which achieved superior performance for older adults.

In order to identify AD and dementia, Buvaneswari et al.^38^ proposed a deep learning technique that uses SegNet to segregate brain features from structural MRI. The method improves the classification of AD, moderate cognitive impairment, and cognitive normal by detecting small morphological alterations. ResNet-101 classifier trained using features taken from 240 sMRI scans makes automated AD diagnosis possible. Goyal et al.^39^ diagnosed Alzheimer’s using LSTM and transfer-learned AlexNet. Their multi-layer system was used to classify MRIs into binary and multiclass categories. To improve the classification results, the authors discovered that their model needed a larger training dataset. They used a GAN to enhance the training dataset in order to address this issue. Their framework performed better than other models, according to a comparison examination.

Kadri et al.^40^ proposed two innovative methods for diagnosing AD. The first method combined a Swin transformer with an improved version of EfficientNet, which includes a Depthwise Over-Parameterized Convolutional Layer (DO-Conv) and multi-head attention. The second method integrated inverted residual blocks and incorporated ECA-Net into the CoAtNet network. Evaluations were conducted using the OASIS and ADNI datasets, while Gradient-based Localization (Grad-CAM) was employed for analysis.

Shah et al.^41^ introduced the Bi-Vision Transformer (BiViT) architecture for classifying various stages of AD and other cognitive disorders using 2D MRI imaging data. To improve feature learning, the BiViT model employs a dual strategy that combines concurrent coupled encoding and mutual latent fusion. Arafa et al.^42^ implemented two methods for analyzing AD. A basic CNN architecture was used in the first approach, while a pre-trained VGG16 model that had been refined by transfer learning on several datasets was used in the second. With fewer labeled training samples and less domain knowledge needed, the suggested method shows mastery of AD analysis and produces notable performance improvements in all diagnostic groups during the experiments. EL-Geneedy et al.^43^ developed a deep learning-based pipeline for the accurate diagnosis and stratification of AD stages. The pipeline provides a quick and accurate AD diagnostic module by using 2D T1-weighted MRI brain images with a shallow CNN architecture. It offers both local classification, which tackles the difficult problem of classifying mild cognitive impairment into Very Mild Dementia, Mild Dementia, and Moderate Dementia as prodromal phases of AD, and global classification (normal vs. Mild Cognitive Impairment vs. AD). In addition to MRI and CT image data taken from the brain, EEG signals are also used to diagnose Alzheimer’s disease. Sercek et al.^44^ introduced a novel graph theoretic, and quantum inspired approach to feature extraction, contributing an innovative perspective to Alzheimer’s diagnosis. In^45^, also the authors have employed biologically inspired EEG analysis, offering a promising noninvasive model grounded in primate brain activity patterns.

This study presents a practical deep-learning approach for predicting Alzheimer’s Disease (AD) using transfer learning and ensemble learning techniques. We fine-tuned three advanced pre-trained models—InceptionResNetV2, InceptionV3, and Xception—for multi-class classification. The final layers of each model were adapted to classify MRI images into four categories: Non-Demented, Very Mildly Demented, Mildly Demented, and Moderately Demented. To improve the prediction performance, we applied an ensemble learning strategy. Each model was trained independently, and their outputs were combined using a majority voting method. This ensemble approach increases the robustness and accuracy of the classification, providing more stable and reliable results than using a single model.

The main contributions of this study are summarized as follows:

- Introducing a robust ensemble framework that combines transfer learning with a majority voting mechanism for accurate classification of Alzheimer’s disease stages.
- Utilizing transfer learning to extract optimal features for multi-class prediction of Alzheimer’s disease by adapting pre-trained models, including InceptionResNetV2, InceptionV3, and Xception.
- Adjusting the final layers of models to improve performance for task-specific classification through fine-tuning.
- Enhancing the model’s efficiency for early prediction of 4-class Alzheimer’s disease, focusing on improvements in accuracy and precision.

## Dataset description

**Fig. 1:**
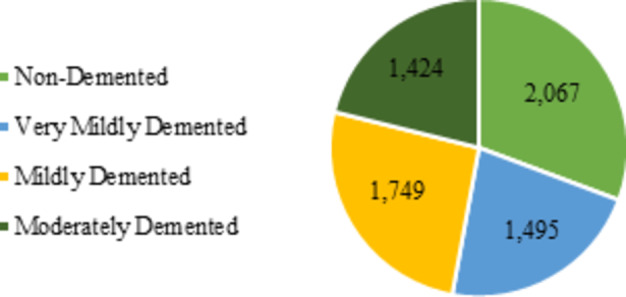
Distribution of samples across classes.

In this study, we utilize the Alzheimer’s Disease Neuroimaging Initiative (ADNI) dataset, which contains 6,735 images categorized into four different cognitive classes: Non-Demented, Very Mildly Demented, Mildly Demented, and Moderately Demented^46^. The ADNI dataset is a comprehensive, multimodal collection that includes not only MRI images but also cerebrospinal fluid (CSF) biomarkers, blood tests, RNA, urine tests, and omics data (encompassing genomics, proteomics, metabolomics, and lipidomic). However, the scope of this study is specifically focused on diagnosing Alzheimer’s disease stages using structural MRI data, and thus, other modalities were not incorporated. Figure 1 shows the distribution of samples across these classes.

Non-Demented class includes 2,067 samples of individuals who exhibit normal cognitive function without any discernible impairment. This group’s participants act as a control population, which is crucial for creating benchmarks that other classes can be measured against. Neuroimaging tests in this group show healthy brain architecture and functions, and they usually don’t exhibit any indicators of cognitive deterioration. Very Mildly Demented consists of 1,495 samples. Participants in this category exhibit early signs of cognitive decline, often characterized by mild memory issues that do not significantly disrupt their daily functioning. Neuroimaging may show subtle changes in brain structure or function, indicating the beginning of neurodegeneration.

**Fig. 2:**
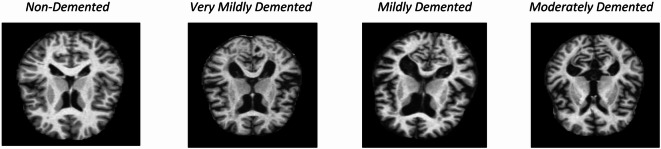
Sample MRI images from the four cognitive classes of the ADNI dataset.

The Mildly Demented class includes 1,749 samples. Individuals in this group display more noticeable cognitive impairments but still retain some level of independence, even though these impairments impact their daily tasks. Common complaints in this category include increased memory loss and difficulties with complex tasks. This group is crucial for studying the progression of AD, as neuroimaging often reveals more apparent atrophy in specific brain regions, such as the hippocampus, which is associated with memory and learning. The Moderately Demented class includes 1,424 samples of individuals with moderate cognitive decline, which significantly impacts their ability to perform daily activities. Participants may experience confusion, memory loss, and difficulty recognizing familiar faces. Neuroimaging data often reveal changes in functional connectivity and significant brain atrophy. To understand the later phases of Alzheimer’s disease and the associated neurobiological changes, it is essential to study this group of individuals. Figure 2 shows sample images from each of the four data classes.

After preprocessing and balancing the data, as well as applying data augmentation techniques, the dataset is divided into three sets: 70% for training, 20% for testing, and 10% for validation. This method enables a thorough analysis of cognitive decline and the progression of AD. Our deep learning model supports known biomarkers, established in prior research, by detecting structural patterns in MRI images. This capability aids in understanding neurobiological changes associated with each class and can facilitate early disease diagnosis.

Our focus was on extracting structural features using convolutional neural network (CNN) models, and thus, demographic data such as age and sex were not included, as they would require a multimodal approach beyond the scope of this research.

## Background

In this study, three models— Inception ResNetV2, InceptionV3, and Xception —were employed to detect Alzheimer’s disease using MRI images. These images provide detailed information and complex features that are crucial for accurately diagnosing this disease. All three models incorporate advanced blocks, such as Inception Modules and Residual Connections. These models possess a more sophisticated architecture than basic deep learning models, enabling them to extract both low-level features, like edges, and high-level features, such as complex shapes, from images simultaneously. This ability enhances their effectiveness in detecting changes in brain structure associated with Alzheimer’s disease.

Another reason for utilizing these models is their low risk of overfitting, due to the presence of mechanisms such as Batch Normalization and Dropout in these models. The architecture of these models is described in more detail below.

### Inception ResNetV2

**Fig. 3:**
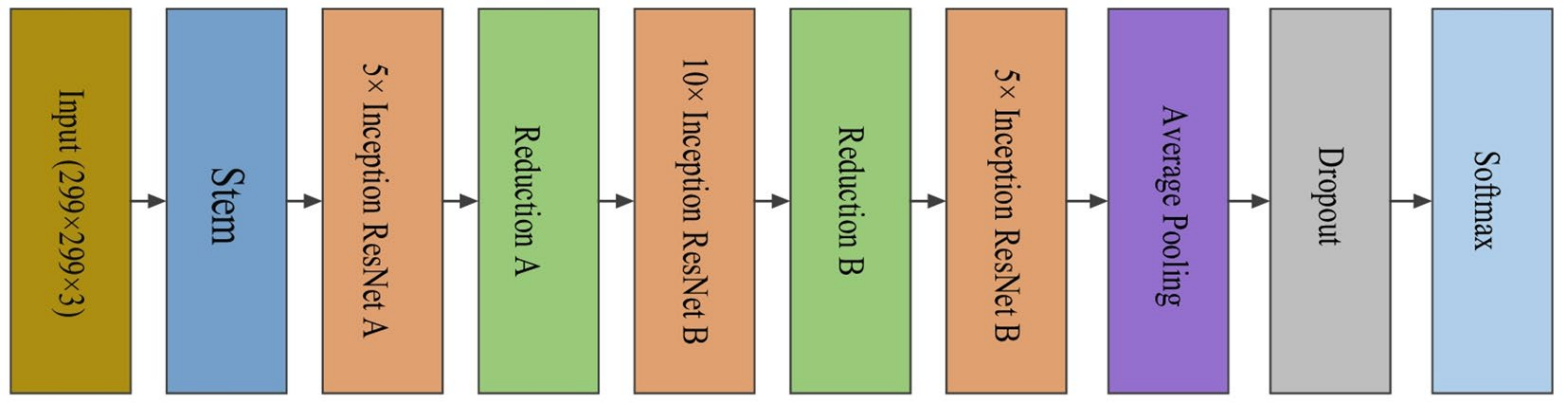
Inception ResNetV2 architecture, featuring Stem (input preprocessing), Inception ResNet blocks (feature extraction), and Reduction modules (dimensionality reduction).

In order to increase accuracy and decrease training time, Szegedy introduced this architecture at Google in 2016, which was trained on over a million images from the ImageNet database^47^. It combines two cutting-edge deep neural network techniques, the Inception and ResNet designs. The architecture consists of Inception blocks that use short ResNet connections. This combination allows the network to efficiently extract features simultaneously and avoid the learning problems of deep networks. The general scheme of this architecture is shown in Fig. 3, which includes the following components^48^:

- *Stem*: This section is positioned at the beginning of the network and is responsible for preparing the inputs for further processing. It includes the initial layers that help effectively distribute features and enable the network to quickly extract information.
- *Inception ResNet A*,* B*,* and C*: These three sections contain Inception blocks that utilize ResNet skip connections, which assist in identifying features in the initial, middle, and final layers.
- *Reduction A*: This section is tasked with reducing the dimensionality of features. This section includes layers that facilitate down-sampling of the input size while maintaining important features. As a result, the network may go deeper without losing important data.
- *Reduction B*: It works like Reduction A, but is used in the later stages of the network. It is designed to reduce dimensionality while preserving features, enabling the network to extract additional features in the final stages.

### InceptionV3

The InceptionV3 model, developed by Google, features multiple parallel Inception blocks. As the third version of the Inception models, Google developed the InceptionV3 architecture, which is made up of multiple parallel Inception blocks^49^.

- Inception A: Contains parallel $\:1\times\:1$ and 3$\:\times\:3$ kernels.
- Inception B: Uses $\:1\times\:1$, 3$\:\times\:3$ and 5$\:\times\:5$ kernels. To improve extraction of more complex features.
- Designed for use in later network stages, this concept is comparable to Inception B.

InceptionV3 also includes down-sampling layers that help reduce the size of features and preserve important information. Additionally, normalization layers aid in increasing learning speed and training stability, which enables the model to train more quickly and precisely^50^.

### Xception

**Fig. 4:**
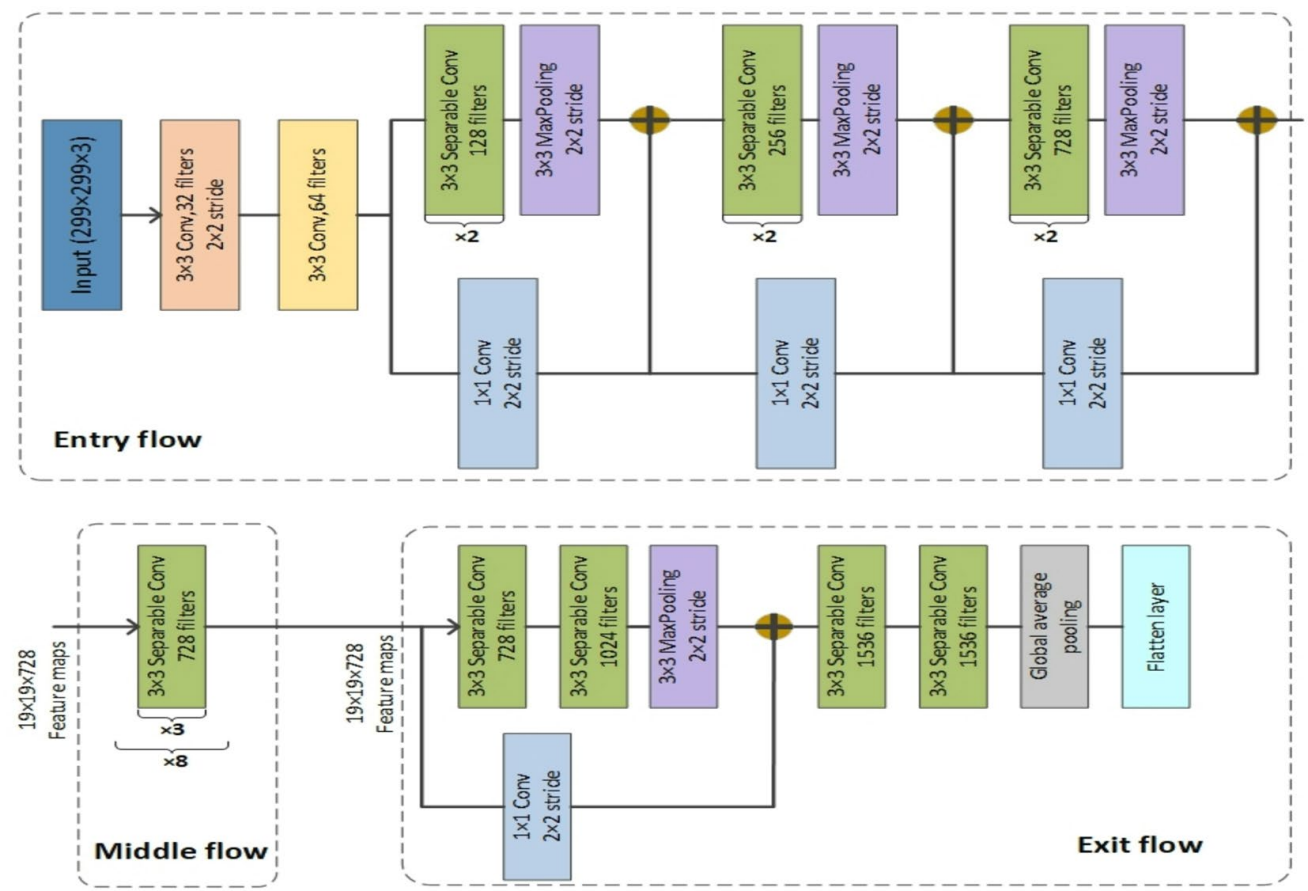
Xception architecture.

Xception, introduced by Francois Chollet at Google, is a deep learning model that extends and complements Inception^51^. In Xception, inception modules are replaced with depthwise separable convolution layers, and the model has a total of 36 layers. Comparing the Xception model with the Inception V3 model, the model performs better on the ImageNet dataset, however, on a larger dataset consisting of 350 million images, Xception performs significantly better. Depthwise separable convolutions comprise the core of the Xception model. In this approach, depthwise convolution is performed first, followed by pointwise convolution. The Xception model employs various $\:n\times\:n\:$filters for depthwise convolution, which occurs after the initial pointwise convolution ($\:1\times\:1$). Figure 4 depicts the Xception model’s architecture and different layers. According to this image, the Xception architecture has three primary components: the entry flow, the middle flow, and the exit flow, with skip connections around the 36 layers^52^.

## Proposed approach

In this study, we emphasize the critical importance of early diagnosis of Alzheimer’s disease, as it can significantly reduce the challenges associated with its progression. Timely intervention can lead to better symptom management and improve quality of life.

**Fig. 5:**
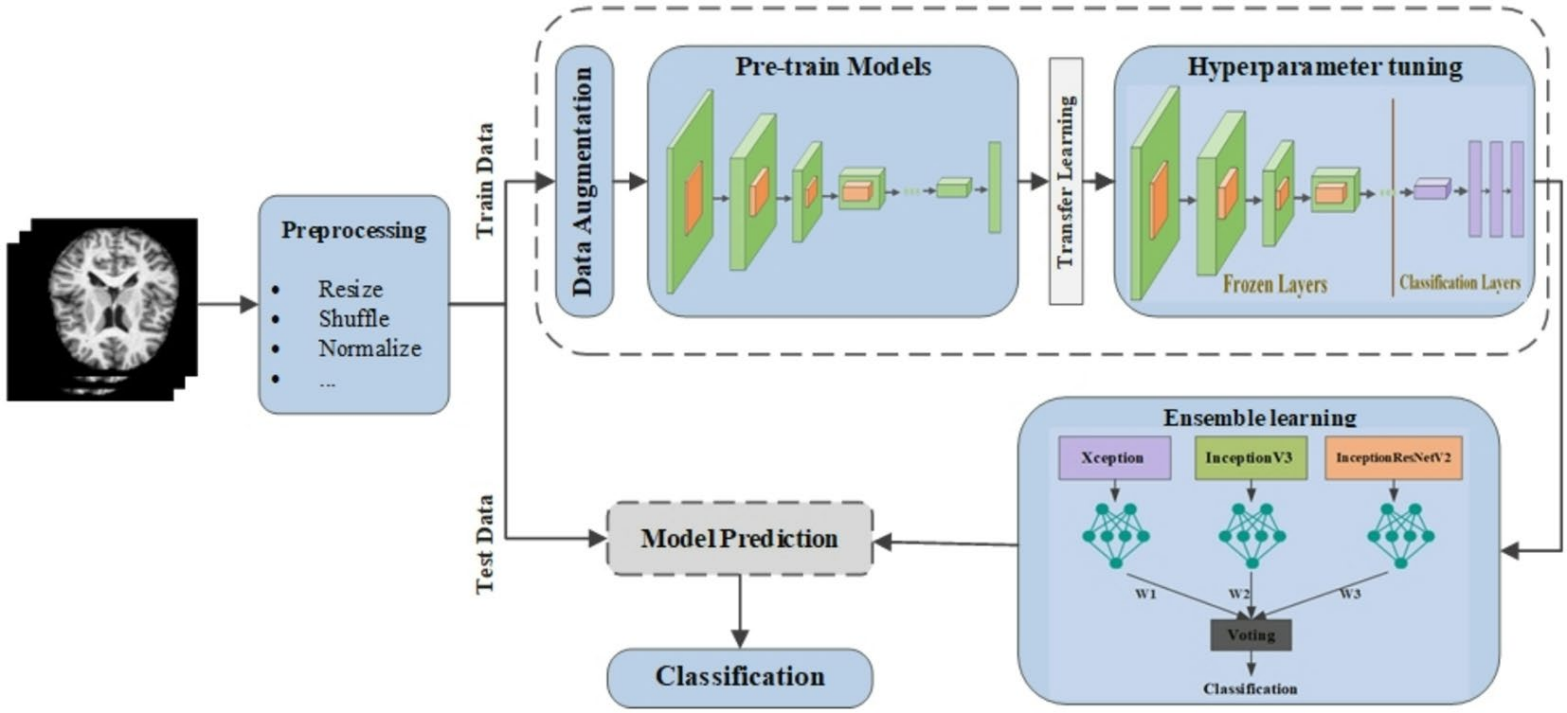
An Overview of the proposed model for early Alzheimer’s diagnosis, featuring preprocessing, training using pre-trained CNN models (InceptionResNetV2, InceptionV3, Xception), hyperparameter tuning and ensemble learning with voting method.

Our Proposed solution consists of several key steps. First, we gather the relevant data on Alzheimer’s disease and then preprocess it to ensure it is suitable for analysis. We apply data augmentation techniques to increase the dataset’s diversity, which enhances the model’s robustness. For classification, we employ the InceptionResnetV2, InceptionV3, and Xception architectures as backbone, known for its improved accuracy and efficiency due to its optimized depth and width. This approach allows us to systematically categorize Alzheimer’s data into four distinct classes. Finally, we thoroughly evaluate the results to assess the effectiveness of our proposed methodology. Figure 5 illustrates an overview of the proposed model.

### Preprocessing

In this study, we utilized the ADNI dataset, which consists of 6,735 samples. To prepare this data for more effective analysis, we implemented a series of preprocessing steps. Initially, each image ***I*** were resized to a uniform dimension of 299 × 299 pixels as indicated in Eq. 1. This standardization is crucial for ensuring compatibility with the InceptionResnetV2, InceptionV3, and Xception architectures.

1 $$\:{I}_{resized}=Resize\:(I,\:(\text{299,299}\left)\right)$$

To tackle data imbalance, we enhanced the dataset using techniques like horizontal flipping with a 90% probability (see Eq. 2) and zooming within ± 20 degrees (see Eq. 3).

2 $$\:{I}_{flipped}=RandomHorizontalFlip\:\left(I,\:p\right)\:\:\:\:\:where,\:p\:=\:0.9$$

3 $$\:{I}_{Zoomed}=Zoom\:\left(I,\:\theta\:\right)\:\:\:\:\:\:\:\:\:\:\:\:\:\:where,\:\theta\:\:=\:20$$

The $\:\text{s}\text{h}\text{e}\text{a}\text{r}\_\text{r}\text{a}\text{n}\text{g}\text{e}$ was set to 0.2, where the transformation is defined as:

4 $$\:\left[\begin{array}{c}x{\prime\:}\\\:y{\prime\:}\end{array}\right]=\:\left[\begin{array}{cc}1&\:shear\_x\\\:shear\_y&\:1\end{array}\right]\left[\begin{array}{c}x\\\:y\end{array}\right]$$

with $\:\text{s}\text{h}\text{e}\text{a}\text{r}\_\text{x}$ and $\:\text{s}\text{h}\text{e}\text{a}\text{r}\_\text{y}$ randomly selected in the range [− 0.2,0.2]. The variables $\:x$ and $\:y$ denote the original pixel coordinates in the image, while $\:x{\prime\:}$ and $\:y{\prime\:}$ indicate the new pixel coordinates after the shear transformation is applied. Images were also randomly shifted by up to 5% of their width and height to introduce spatial variability and improve model robustness.

**Fig. 6:**
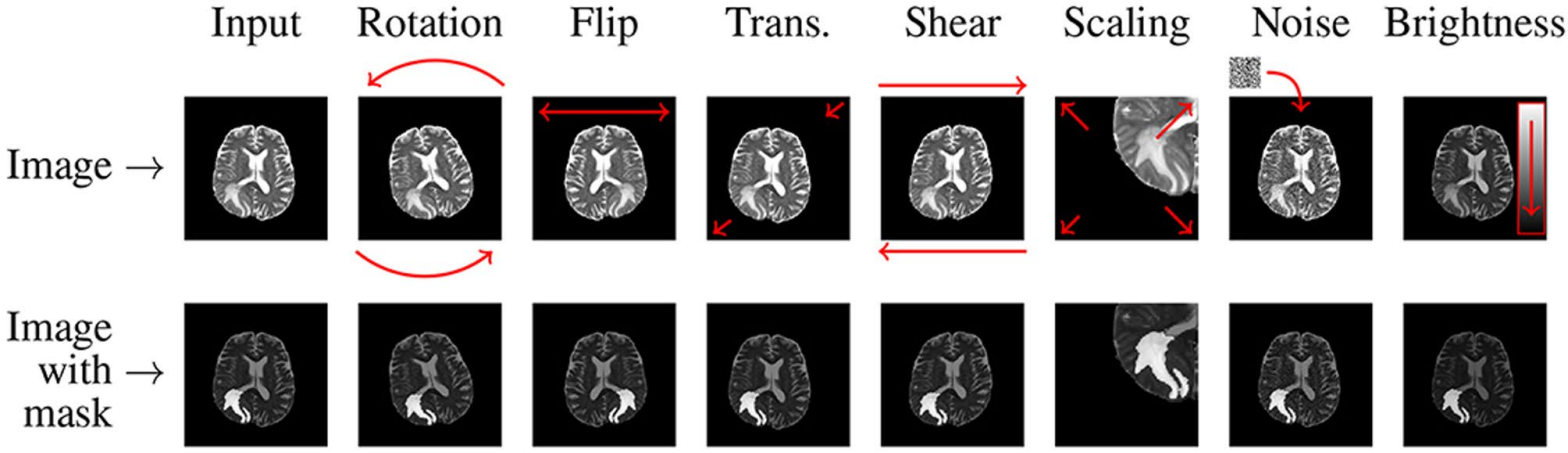
Data augmentation methods applied on an image.

Data augmentation not only increases the diversity of the training samples but also improves the model’s ability to generalize unseen data, helping to mitigate the overfitting problem. Figure 6 shows some images created by augmentation techniques.

Additionally, all images were converted from color (RGB with three channels) to grayscale (single channel) as shown in Eq. 5. This conversion aimed to simplify the model and reduce complexity while retaining essential features. After converting to grayscale, pixel values were normalized from a range of 0-255 to 0–1, following Eq. 6. This normalization aligns the data with the input requirements of the pre-trained InceptionResnetV2, InceptionV3, and Xception architectures, ensuring consistent and high-quality input for accurate classification.

5 $$I\,=\,0.{\text{299}} \times R\,+\,0.{\text{587}} \times G\,+\,0.{\text{114}} \times B$$

6 $$\:{I}_{norm}=I/255\:\:$$

### Architecture

The proposed architecture combines the strengths of the three architectures mentioned above along with transfer learning and layer customization for the last layers. All three models were originally trained on the large ImageNet dataset, which helps them learn general image features. In our approach, the base layers of each model are kept frozen, and only the final classification layers are trained on the Alzheimer’s dataset. This helps the models focus on learning task-specific patterns without losing the powerful feature extraction abilities from the pre-trained layers. This approach effectively combines transfer learning and hyperparameter tuning, employing the general feature extraction capabilities of pre-trained models along with task-specific adjustments made through hyperparameter tuning.

All three architectures begin with a 2D convolution layer to preprocess the input data, followed by the model seen from the pre-training. GlobalAveragePooling2D and Flatten layers were added to reduce the spatial dimension and transform the multi-dimensional features into a 1D vector, respectively. Fully connected (Dense) layers with ReLU activation functions are incorporated to enhance the model’s ability to learn complex patterns. Dropout layers are added to mitigate overfitting. Finally, a Dense layer with four units and a softmax activation function is included to generate the output probabilities for the four target classes. The key parameters and configurations utilized in our proposed architecture are presented in Table 1.

**Table 1:** Overview of main parameters for the base and proposed models used in alzheimer’s diagnosis.

Parameter	Value/configuration
Base Models	InceptionResNetV2, Xception, InceptionV3
Input Shape	(229, 229, 3)
Dropout Rate	0.3
Learning Rate	0.003
Optimizer	Adam
Epochs	30
Batch size	16
Loss Function	Sparse categorical cross entropy
Evaluation Metric	Accuracy

**Fig. 7:**
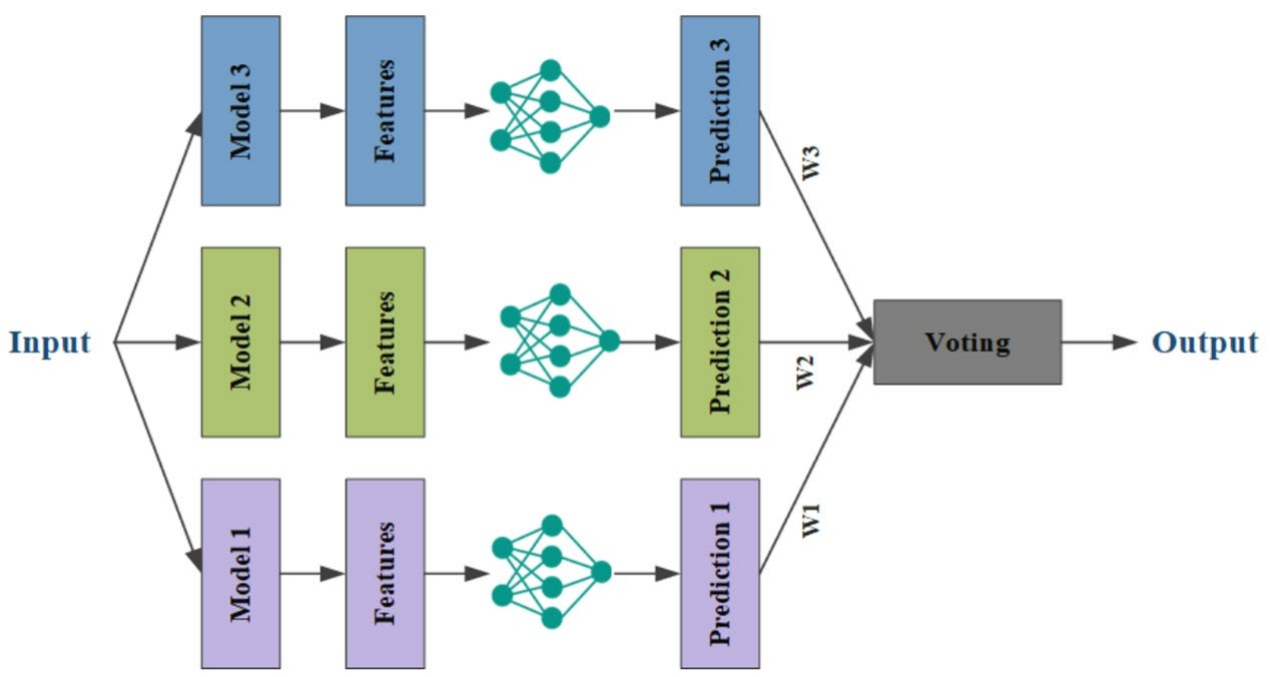
Weighted ensemble learning method for classifying Alzheimer’s disease, combining votes from InceptionV3, Xception, and InceptionResNetV2 with weights based on performance.

To enhance the efficiency of the proposed models, we employed an ensemble learning approach that combines the predictions from three different architectures using a voting mechanism. Figure 7 illustrates the overall method of weighted ensemble learning. This method not only strengthens the model’s performance but also minimizes the risk of overfitting and improves generalization. In general, ensemble learning uses the strategy of effectively combining different individual learners. Each individual learner is generated from training data by existing learning algorithms. Using individual learners of the same type is called homogeneous ensemble learning, and using various individual learners is called heterogeneous ensemble learning. In this research, homogeneous ensemble learning is used by combining CNN networks including InceptionV3, Xception, InceptionResNetV2.

There are three strategies for combining models, which include voting, averaging, and stacking. In this research, the voting method is used. This method assumes that there are *T* different classifiers including ℎ_1_, ℎ_2_, …, ℎ_T_. The objective is to use the classifier’s output to forecast the final category from the category markers *c*_1_, *c*_2_, ….*c*_*l*_. The output result of the classifier ℎ_i_ for a sample *x* is usually a one-dimensional label vector $\:{({h}_{i}^{1}\left(x\right),{h}_{i}^{2}\left(x\right),\dots\:,{h}_{i}^{l}\left(x\right))}^{T}$, where $\:{h}_{i}^{j}\left(x\right)$ is the prediction output of the ℎ_*i*_ classifier on the class *c*_*j*_ label.

The proposed ensemble learning method assigns a weight to each network based on its performance. If a classifier performs better, it is assigned a higher weight. First, the weights are scaled to values between 0 and 1. Then, for each weight vector, the combination of weights that has the highest accuracy in predicting the disease stages is selected. These optimized weights are applied in the final stage of the ensemble learning method. The weighted ensemble learning formula is given in Eq. (7):

7 $$\:H\left(x\right)=\:{c}_{{arg}_{j}max}\:\sum\:_{i=1}^{T}{w}_{i}{h}_{i}^{j}\left(x\right),\:$$

where the weight of classifier ℎ_*i*_ is represented by $\:{w}_{i}$. In real-world applications, the weight coefficients are frequently normalized and restricted by $\:{w}_{i}\ge\:$ 0 and $\:\sum\:_{i=1}^{T}{w}_{i}=1$, much like in the weighted average approach. Selecting the appropriate weight is crucial. Selecting the appropriate weights for classifiers is crucial and should align with the performance of each individual classifier, provided that the output of each classifier operates independently from the others.

## Experimental results and model evaluation

The experiments were conducted to implement the models in a Google Colab environment, utilizing a system with an Intel Core i7 processor, 16 GB of RAM, and running on Windows 11.

We compared the performance of three transfer learning models InceptionResNetV2, InceptionV3, and Xception for Alzheimer’s disease classification across four categories. Following this, we benchmarked these models against the proposed ensemble learning model to evaluate its effectiveness in improving diagnostic accuracy. This comparative analysis enables us to assess the advantages of using these models in the context of Alzheimer’s disease classification. For the evaluation of these models, we employed several metrics to provide a thorough assessment of their performance^53^. The key evaluation criteria include Accuracy, Precision, Recall, and F1-Score. In addition, the confusion matrix offers a detailed view of model performance, allowing for a detailed analysis of classification errors^54^.

### Model evaluation

The results presented in this subsection were obtained by evaluating the proposed deep learning model on the test split of the ADNI dataset. The model was trained on all four classes (Non-Demented, Very Mildly Demented, Mildly Demented, and Moderately Demented) and evaluated on a separate test set, comprising 20% of the data. Training curves, including learning curves, demonstrate the model’s stable performance and absence of overfitting, confirming the validity of the results.

Table 2 presents a performance comparison of the proposed methods, focusing on overall evaluation metrics. This table displays the average accuracy, precision, recall, and F-score across all classes. As shown, Inception ResNetV2 and Ensemble Voting are the top-performing models, followed closely by Xception, which ranks third. InceptionV3 also shows good results, although it performs slightly weaker than the other models. Table 3 presents the performance of the models according to various evaluation criteria, including Accuracy, Precision, Recall, and F-Score, across different data classes: Non-Demented, Very Mildly Demented, Mildly Demented, and Moderately Demented. All models demonstrated high accuracy in diagnosing Alzheimer’s disease; however, Inception ResNetV2 model proposed in this study exhibited the highest accuracy as well as Ensemble voting. In the Non-Demented category, both Inception ResNetV2 and Ensemble voting outperformed other models in terms of all metrics. Notably, the Recall criterion for the Ensemble voting model was also superior to that of the other models, achieving a value of 0.98 and the precision criterion for the Inception ResNetV2 model was also superior to that of the other models, achieving a value of 0.97.

**Table 2:** Performance comparison of the proposed methods in terms of evaluation metrics.

Architecture	Accuracy	Precision	Recall	F-score
Inception ResNetV2	0.96	0.97	0.96	0.96
InceptionV3	86.46	0.88	0.86	0.86
Xception	0.93	0.93	0.93	0.93
Ensemble voting	0.98	0.98	0.98	0.98

All models performed exceptionally well in diagnosing Mildly Demented patients, with a Recall criterion of 1.00, but Xception model with 0.99. Among these models, Inception ResNetV2 and Ensemble voting were identified as the top-performing options. In general, all models showed slightly reduced performance in detecting the Very Mildly Demented class compared to the other classes, with Inception ResNetV2 being the least effective. However, the Ensemble voting model excelled in this area, achieving a Precision of 0.97 and a Recall of 0.95. On the other hand, all models performed exceptionally well in identifying the Moderately Demented class, with Precision, Recall, and F-Score metrics all reaching 1.00 for each model.

**Table 3:** Class-wise performance comparison of each class for the proposed methods in terms of evaluation metrics.

Class	Architecture	Precision	Recall	F-Score
Non-demented	Inception ResNetV2	0.97	0.97	0.97
	InceptionV3	0.93	0.69	0.79
	Xception	0.91	0.89	0.90
	Ensemble voting	0.96	0.98	0.97
Mildly demented	Inception ResNetV2	1.00	1.00	1.00
	InceptionV3	0.87	1.00	0.93
	Xception	0.97	0.99	0.98
	Ensemble voting	1.00	1.00	1.00
Very mildly demented	Inception ResNetV2	0.96	0.95	0.95
	InceptionV3	0.69	0.82	0.75
	Xception	0.86	0.85	0.85
	Ensemble voting	0.97	0.95	0.96
Moderately demented	Inception ResNetV2	1.00	1.00	1.00
	InceptionV3	1.00	1.00	1.00
	Xception	1.00	1.00	1.00
	Ensemble voting	1.00	1.00	1.00

**Fig. 8:**
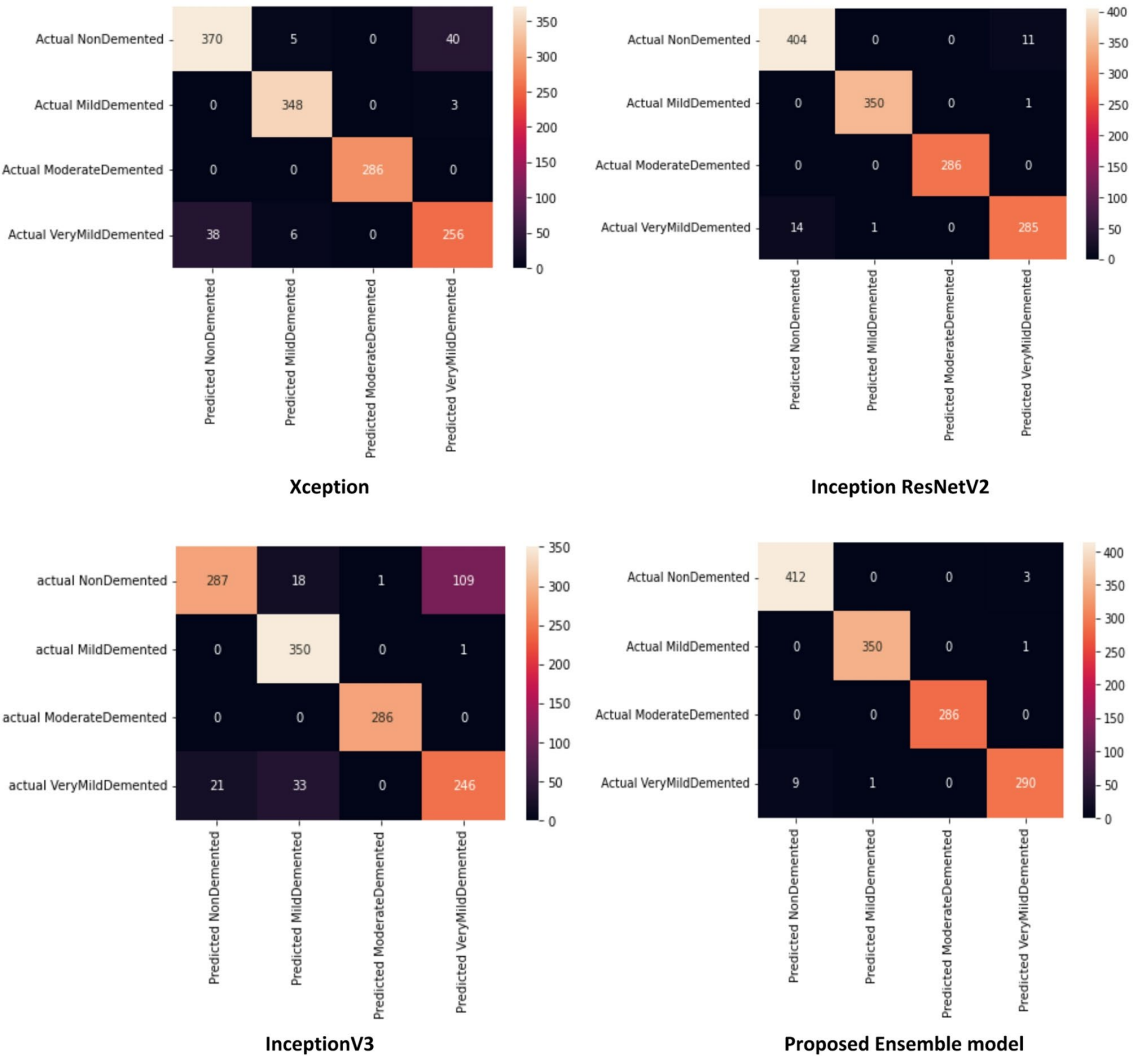
Confusion matrix of the base and proposed ensemble models for classifying four Alzheimer’s classes (Non-Demented, Very Mildly, Mildly, Moderately) with ADNI MRI images.

Subsequently, the four proposed models were compared using their confusion matrices, as illustrated in Fig. 8.

The Xception model demonstrated perfect classification accuracy for the moderated demented class, with zero misclassifications. However, its primary errors occurred in the nondemented group. It identified 38 samples related to the very mildly demented class as nondemented and also incorrectly classified 45 samples that were nondemented into other categories. The Inception ResNetV2 model also has the highest error in detecting nondemented. In contrast, InceptionV3 as the most week model has the highest misclassification of very demented samples as nondemented to 21 instances and incorrectly classified 128 samples that were nondemented into other categories. Notably, the ensemble learning model achieved superior performance across all classes, committing only 9 misclassifications of very demented samples as nondemented—the lowest among all evaluated architectures. A consistent pattern across all models was the misclassification of very demented samples into the nondemented category. Misclassifications between control (Non-Demented) and Very Demented samples in Fig. 8, though surprising, can occur in deep learning models, particularly for complex tasks like Alzheimer’s disease diagnosis. These errors may stem from factors such as slight class imbalance (e.g., fewer Very Demented samples compared to Non-Demented in the ADNI dataset), noise in MRI data, or limitations in the model’s ability to distinguish subtle features in early disease stages.

Overall, the numerical results, along with confusion matrix plots, indicate good performance of the models. Additionally, the classification performance of the ensemble learning method was generally superior to that of the individual transfer learning methods. The proposed model was finally compared with four other state-of-the-art methods concerning accuracy metric. The results of this comparison are presented in Table 4. Saratxaga et al.^55^ employed three different models—ResNet18, BrainNet2D, and BrainNet3D—to predict Alzheimer’s disease based on brain MRI data. However, the accuracy of these models was lower than other competitors. Jabason et al.^56^ utilized a hybrid ensemble model to classify Alzheimer’s disease from MRI data, achieving an accuracy of 0.95. Sarraf and Tofighi^57^ used the LeNet-5 model for this classification, which yielded an acceptable accuracy of approximately 0.96. Cui et al.^58^ developed an enhanced inception network based on brain MRI to diagnose Alzheimer’s disease, achieving an accuracy of 0.85. In contrast, our proposed method outperformed all other models, achieving an accuracy of 0.98.

**Table 4:** Accuracy comparison of the proposed ensemble voting model with other state-of-the-art models.

Reference	Data type	Model		Accuracy
Saratxaga et al.^55^	MRI		ResNet18	0.88
			BrainNet2D	0.85
			BrainNet3D	0.77
Jabason et al.^56^	MRI		Hybrid deep learning	0.95
Sarraf and Tofighi^57^	FMRI		LeNet-5	0.96
Cui et al.^58^	MRI		InceptionV3	0.85
**Proposed model**	**MRI**		**Ensemble voting**	**0.98**

### Ablation study

To thoroughly evaluate the effectiveness of our proposed ensemble framework, we conducted an ablation study to evaluate the individual contributions of each base model (InceptionResNetV2, InceptionV3, and Xception) and their combinations. This analysis helps isolate the impact of different architectural choices and ensemble strategies on the overall performance. Table 5 shows the performance of different ensemble models with respect to the Precision, Recall, F-Score, and Accuracy metrics.

**Table 5:** Performance comparison of different ensemble methods in terms of evaluation metrics.

Architecture	Accuracy	Precision	Recall	F-score
Inception ResNetV2 & InceptionV3	0.96	0.96	0.96	0.96
Inception ResNetV2 & Xception	0.97	0.97	0.97	0.97
InceptionV3 & Xception	0.93	0.93	0.93	0.93
All models	0.98	0.98	0.98	0.98

As you can see in the table of results of ensemble different models, better results are obtained when all three architectures are combined. After that, the combination of Inception ResNetV2 & Xception performs better than the others. Since the InceptionV3 model performs much weaker than the others, ensemble can well have a positive and significant impact on its performance, as it performs much better after combining with Xception.

## Limitations and future work

Although our proposed deep learning framework demonstrates promising results for the early and accurate classification of Alzheimer’s disease stages, several limitations must be acknowledged to contextualize its findings. The dataset used in this study, although standardized and collected with high accuracy, has limitations that could limit the model’s ability to generalize across the full spectrum of disease variability. In addition, variations in MRI imaging protocols, such as differences in scanner types, imaging parameters, or imaging resolution across institutions, may introduce biases that affect the consistency of image quality and, consequently, model performance. Demographic factors, including age, ethnicity, sex, or socioeconomic status, may also contribute to biases if the training data lacks adequate representation of diverse populations. The model should also be validated in real-world clinical settings, which are crucial to ensure its applicability in routine medical settings. Finally, the inherent lack of interpretability in deep learning models may reduce clinician confidence, as the decision-making process remains ambiguous without explicit links to neuroanatomical or pathological correlates.

To address these limitations, future research should prioritize model validation on larger and more diverse datasets collected from multiple institutions to increase generalizability and reduce biases due to imaging variations or population imbalances. Standardization of MRI imaging protocols or application of advanced data augmentation techniques could further improve the robustness of the model to image quality inconsistencies. Furthermore, the inclusion of multimodal data, such as clinical assessments and genetic markers, could provide a more comprehensive approach to Alzheimer’s diagnosis and potentially improve predictive accuracy and clinical relevance. Prospective validation studies in real-world clinical settings, along with user studies involving neurologists to assess model usability and interpretation, are essential to bridge the gap between research and clinical practice. Techniques from explainable AI (XAI), such as attention maps or saliency visualizations, should be incorporated to highlight disease-relevant regions and align model outputs with clinical understanding.

## Conclusion

In this study, an advanced CNN framework for the early and accurate multi-class classification of Alzheimer’s disease was proposed. This framework leveraged transfer learning, hyperparameter tuning, and ensemble voting to address the challenges of diagnosing AD across its progressive stages. By adapting state-of-the-art architectures—InceptionResNetV2, InceptionV3, and Xception—and optimizing their final layers to predict four categories: Non-Demented, Very Mildly Demented, Mildly Demented, and Moderately Demented, we achieved significant improvements in diagnostic performance. Outperforming all individual baseline models, the ensemble voting mechanism, which included predictions from various models, showed remarkable robustness, attaining 98.96% accuracy and 100% precision for crucial classes (Mildly and Moderately Demented). Additionally, the InceptionV3 model emerged as a strong candidate for standalone classification. Overall, the results of this research have the potential to assist doctors in diagnosing Alzheimer’s disease at an early stage and help patients to the disease’s progression. However, there are still many challenges and ongoing studies that need to be addressed in future research. Incorporating multimodal data and considering other clinical and genetic factors in patients could lead to more precise and robust predictions.

## Data Availability

The dataset analyzed during the current study are available in the https://www.kaggle.com/datasets/praneshkumarm/multidiseasedataset.

## Code availability

The source codes of the current study are available in the https://github.com/muhammadmo/Alzheimer_Classification_MRI.
